# Safety and effectiveness of anticoagulation therapy in older people with atrial fibrillation during exposed and unexposed treatment periods

**DOI:** 10.1136/heartjnl-2024-324763

**Published:** 2025-02-17

**Authors:** Anneka Mitchell, Margaret C Watson, Tomas J Welsh, Anita McGrogan

**Affiliations:** 1 Department of Life Sciences, University of Bath, Bath, UK; 2 Pharmacy Department, Plymouth Hospitals NHS Foundation Trust, Plymouth, UK; 3 Strathclyde Institute of Pharmacy and Biomedical Sciences, University of Strathclyde, Glasgow, Glasgow, UK; 4 The ReMind UK Centre, Royal United Hospital Bath NHS Trust, Bath, Bath and North East Somerset, UK; 5 Institute of Clinical Neurosciences, University of Bristol, Bristol, UK

**Keywords:** ANTICOAGULATION, Atrial Fibrillation, Stroke, Epidemiology, Pharmacology, Clinical

## Abstract

**Background:**

Anticoagulation therapy reduces stroke risk in patients with atrial fibrillation (AF), but it is often underused in older populations due to concerns about bleeding. This study aimed to compare the safety and effectiveness of anticoagulation during periods of exposure and non-exposure and across different anticoagulants in people with AF aged ≥75 years.

**Methods:**

Using UK primary care data from the Clinical Practice Research Datalink (2013–2017), a retrospective cohort study was conducted on patients newly prescribed oral anticoagulants (warfarin or direct oral anticoagulants). Exposure to anticoagulation was mapped using prescription data. Cox regression models were used to estimate adjusted HRs for stroke, bleeding, myocardial infarction, and death during periods of exposure and non-exposure and for different anticoagulants.

**Results:**

Among 20 167 patients (median age 81 years), non-exposure to anticoagulation was associated with higher risks of stroke (HR 3.07, 95% CI 2.39 to 3.93), myocardial infarction (HR 1.85, 95% CI 1.34 to 2.56) and death (HR 2.87, 95% CI 2.63 to 3.12) compared with exposure. Compared with warfarin, apixaban was associated with lower risks of non-major bleeding (HR 0.73, 95% CI 0.64 to 0.85), whereas rivaroxaban was associated with higher risks of major (HR 1.33, 95% CI 1.15 to 1.55) and non-major (HR 1.29, 95% CI 1.16 to 1.44) bleeding.

**Conclusions:**

Non-exposure to anticoagulation increases the risks of stroke, myocardial infarction and death in older patients with AF. Clinicians should carefully weigh the risks of discontinuing anticoagulation and provide shared decision-making support to patients, especially when considering deprescription.

**WHAT IS ALREADY KNOWN ON THIS TOPIC**

- Anticoagulation reduces the risk of stroke in patients with atrial fibrillation (AF) but is often withheld or discontinued in older populations due to bleeding concerns.
- Limited evidence exists on the outcomes of non-exposure or deprescription of anticoagulants in this population.

**WHAT THIS STUDY ADDS**

- Non-exposure to anticoagulation in older adults with AF is associated with significantly higher risks of stroke, myocardial infarction and death, without major reductions in bleeding risks.
- Among direct oral anticoagulants, apixaban showed a lower risk of non-major bleeding, while rivaroxaban was associated with higher bleeding risks compared with warfarin.

**HOW THIS STUDY MIGHT AFFECT RESEARCH, PRACTICE OR POLICY**

- Decisions to discontinue anticoagulation in older patients should consider the significantly increased risks of adverse outcomes during non-exposure periods.
- Apixaban may offer a safer bleeding profile compared with warfarin and rivaroxaban, supporting its consideration as a preferred option in older patients with bleeding concerns.

## Introduction

 Atrial fibrillation (AF) is a common cardiac condition which increases the risk of stroke.^1^ Strokes associated with AF are often more severe and have a higher rate of mortality than those that occur in people without AF;^2 3^ effective stroke prevention is therefore a key component of AF management.

Anticoagulation has been shown to significantly reduce the risk of stroke, particularly in older patients who have the greatest risk.^4^ The direct oral anticoagulants (DOACs), dabigatran, rivaroxaban, apixaban and edoxaban are now recommended as the first-line option for most patients^5 6^ and have largely superseded vitamin K antagonists such as warfarin due to their comparable efficacy and lower risk of adverse events.^7^,^10^

When deciding whether to commence anticoagulation, there is a need for prescribers to balance the risk of side effects from the medication with the risk of stroke associated with not prescribing. Often, prescribers are more concerned about the bleeding risk from the anticoagulants, and warfarin has historically been underprescribed to older people who are at an increased risk of both stroke and bleeding.^11^ While DOACs have a number of advantages over warfarin, older patients and those with a history of falls or frailty are still less likely to be prescribed these medications.^12^,^14^ Studies evaluating patient preferences for anticoagulation have demonstrated that their perception of risk differs to that of prescribers with patients valuing stroke prevention over concerns about bleeding risk.^15^

Clinically, prescribers discontinue anticoagulation for the same reasons.^16^ Patients may also decide to discontinue treatment due to adverse events such as bleeding but patient motivation for stopping treatment is not well documented.^16^ Discontinuation rates for warfarin and dabigatran have been reported to range from 6% to 15% at 2 years^16^ with lower rates reported for apixaban and rivaroxaban.^16^ One study in the UK reported that, in the first year of therapy, 10% of patients stopped DOAC treatment and did not restart, whereas 20% of patients in their cohort discontinued their DOAC for >30 days then restarted.^17^ Anticoagulant withdrawal has been associated with an increased risk of stroke.^18^,^20^

The aim of this study was to compare safety (major and non-major bleeding, myocardial infarction and death) and effectiveness (stroke) outcomes in patients aged ≥75 years with AF in UK primary care who at the time of study entry had been newly started on an anticoagulant. We compared outcomes in people exposed or not exposed to anticoagulant treatment at the time of the event. We also compared outcomes with different anticoagulant exposures.

## Methods

### Study design

We conducted a retrospective cohort study with a new-user design using the UK Clinical Practice Research Datalink (CPRD). The CPRD contains anonymised general practice records for patients in the UK and is representative of the population.^21^ The exposures of interest were dabigatran, rivaroxaban, apixaban, edoxaban or warfarin for stroke prevention in AF. We had full access to the database population used to extract the study population.

### Study cohort

We extracted all patients with a Read code for AF or atrial flutter. Patients included in the study had to be permanently registered with the general practice, have a minimum of two Read codes for AF or one Read code for AF plus supporting evidence, for example, a clinic referral, and had to be prescribed an anticoagulant during the study period (1 January 2013 to 27 December 2017). Patients joined the study on the date of their first prescription for an anticoagulant of interest, defined as the index exposure, and the date of this prescription was defined as the index date. They needed at least 1 year without any prescriptions for an anticoagulant before their index date and could only enter the study once.

Patients were aged ≥75 years on the index date and had a minimum of 1 year of research standard data prior to and 1 month following the index date.

**Figure 1:**
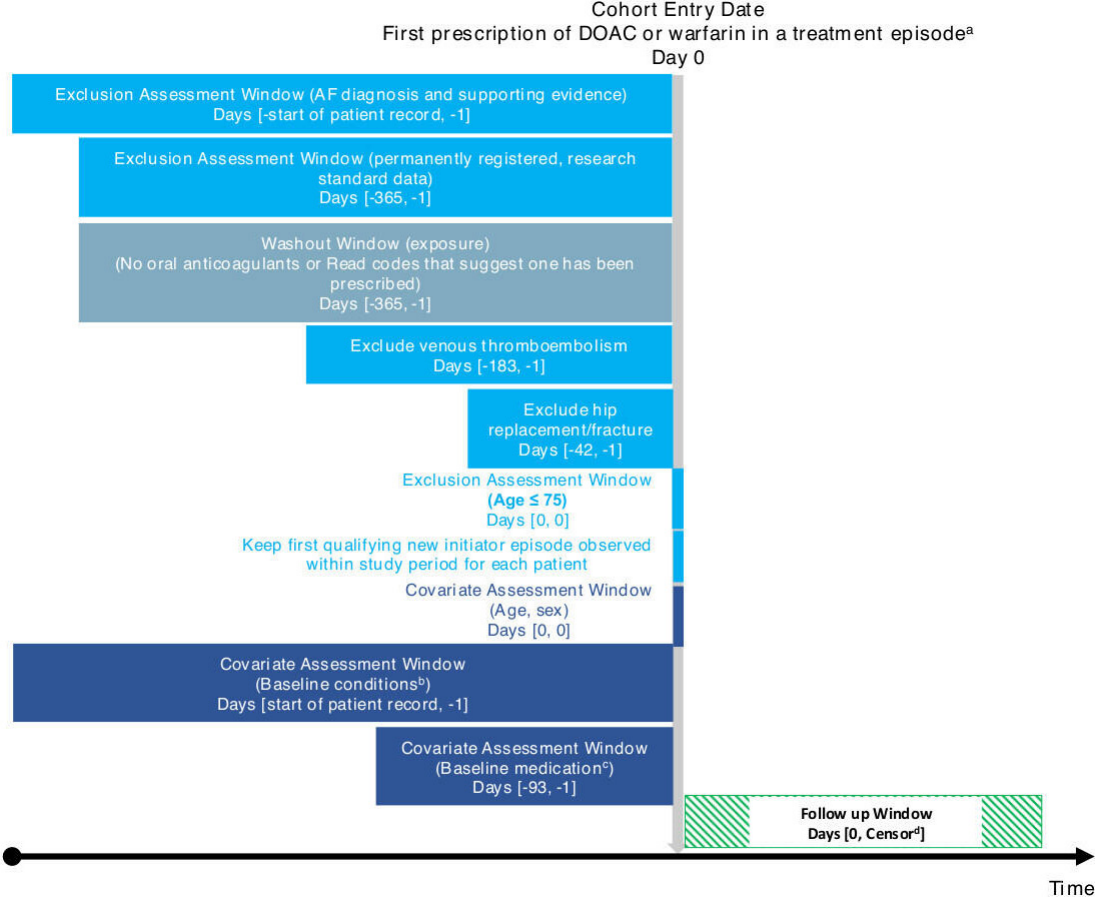
Summary of the study design, criteria for cohort entry, assessment of covariates and follow-up. Time periods are shown as Days (x,y) where x is the start and y is the end of the time period. Day 0 denotes the date of cohort entry. Minus figures indicate the number of days before cohort entry (template from http://www.repeatinitiative.org/projects.html). ^a^Treatment episodes defined by date of prescription and quantity supplied with a stockpiling algorithm if a new prescription occurs before the end of the date supply. For DOACs, gaps of less than 60 days between end date of supply and next prescription were bridged. For warfarin, a gap of twice the median gap between previous prescriptions was allowed. ^b^Baseline condition included: hypertension, heart failure, coronary artery disease, peripheral vascular disease, diabetes mellitus, stroke, transient ischaemic attack, kidney disease, liver disease, bleeds, dementia, falls, fragility fracture. ^c^Baseline medications included: antihypertensives, antiplatelets, non-steroidal anti-inflammatories, corticosteroids, selective serotonin reuptake inhibitors, proton pump inhibitors of H_2_ antagonists, statins, diabetic medications, anticonvulsants, antiarrhythmics. ^d^Earliest of: outcome of interest, death, end of research standard data, end of the study period. AF, atrial fibrillation; DOAC, direct oral anticoagulant. RECORD-PE, REporting of studies Conducted using Observational Routinely-collected Data guidelines extended for PharmacoEpidemiological research.

Figure 1 summarises the key elements of the study design as recommended by the RECORD-PE reporting guidelines (REporting of studies Conducted using Observational Routinely-collected Data guidelines extended for PharmacoEpidemiological research).^22^

### Definition of exposure and non-exposure

Anticoagulant prescription data were extracted from the CPRD for individual patients. Treatment episodes (exposed time) were then constructed for DOACs and warfarin, respectively. Gaps in treatment episodes were classified as unexposed time.

For DOACs, treatment episodes were defined using the quantity prescribed and licensed dose per day (one for rivaroxaban and edoxaban, two for dabigatran and apixaban). Gaps of ≤60 days between the end of one prescription and the start of the next were filled and classed as a continuous treatment episode. If there was a gap of >60 days between the calculated end of the prescription this was defined as the end of that treatment episode.

For warfarin, which does not have a fixed dosing schedule, an algorithm was developed to predict when a treatment episode started and ended. The algorithm used a combination of prescription data (date of prescription, strength and quantity of tablets), gaps between prescriptions and international normalised ratio test results. The algorithm was further improved by including Read codes suggesting that warfarin therapy had either continued or stopped (eg, warfarin contraindicated) (see online supplemental appendix 1 for more detailed methods).

### Outcomes

Outcome events were attributed to the anticoagulant exposure at the time of the event if the event occurred during a treatment episode, or to no exposure if the event occurred in a treatment gap. The primary effectiveness outcome was ischaemic or unspecified stroke (ischaemic or haemorrhagic). The primary safety outcome was major bleeding. Secondary safety outcomes were clinically significant non-major bleeding, intracranial haemorrhage, gastrointestinal bleeding, myocardial infarction and death. Major bleeds were defined as per the International Society of Thrombosis and Haemostasis,^23^ with the addition of gastrointestinal bleeding. Clinically relevant non-major bleeds were any other bleeds recorded that had resulted in medical attention.

### Covariates

Covariates at baseline were determined by the presence of a Read code at any time before the index date. Patients were required to have a minimum of 1 year research standard data prior to study entry to capture covariates. This time frame was chosen as all patients registering with general practice in the UK are required to provide a full medical history and previous studies have shown that incidence rates of diseases recorded in CPRD tend to settle in this time.^24^

Covariates included age at index date, biological sex, smoking status, alcohol intake and relevant comorbidities that could influence development of the outcomes of interest (see online supplemental appendix 1). The level of frailty was calculated using an algorithm based on the electronic frailty index domains.^25^

Prescription information from 3 months prior to the index date was extracted for: antihypertensives, antiarrhythmics, anticonvulsants, diabetes medications, statins, non-steroidal anti-inflammatories, corticosteroids, selective serotonin reuptake inhibitors, proton pump inhibitors and H_2_ antagonists.

Read codes were used to identify the study cohort, covariates and outcomes. The quality of Read code recording in primary care data in the UK is variable as data are collected for the purposes of patient care, not research.^21^ Diagnoses associated with the Quality Outcome Framework are more reliably recorded.^26^

### Statistical analysis plan

Baseline characteristics were reported; person-years of follow-up were calculated from the index date to the earliest date of the outcome or the final recording for the patient.

Survival analysis was used to determine the hazard of each outcome in people exposed and not exposed to anticoagulation at the time of the event. A second analysis compared people exposed to different anticoagulants at the time of the event. Crude HRs were determined using Cox regression models then adjusted for the covariates listed. All covariates were included in the initial model. Any covariates with a value of p>0.2 in the full model were then removed to produce a simplified model. The proportional hazards assumption was tested using Schoenfeld residuals.^27^ The analysis for each outcome was conducted individually and patients were censored on the date of the outcome for that analysis. Patients could therefore contribute to different exposure groups depending on if they were exposed to an anticoagulant and which anticoagulant they were exposed to at the time of the event for that analysis.

### Sensitivity analyses

We conducted an intention to treat analysis to compare anticoagulant treatments where patients were censored at the point of treatment discontinuation or switch, outcome occurrence, or end of study.

We hypothesised that an increased risk of death in treatment gaps may have been attributable to anticoagulation being stopped due to a palliative diagnosis or the patient moving to end-of-life care. We conducted a post hoc analysis and extracted the 100 most frequently recorded Read codes recorded in the month before a treatment gap and the month before death to investigate whether there were any trends.

Statistical analysis was conducted using Stata V.16.

### Patient and public involvement

Patients and the public were not involved in the design, conduct, reporting, or dissemination of this research.

## Results

**Figure 2:**
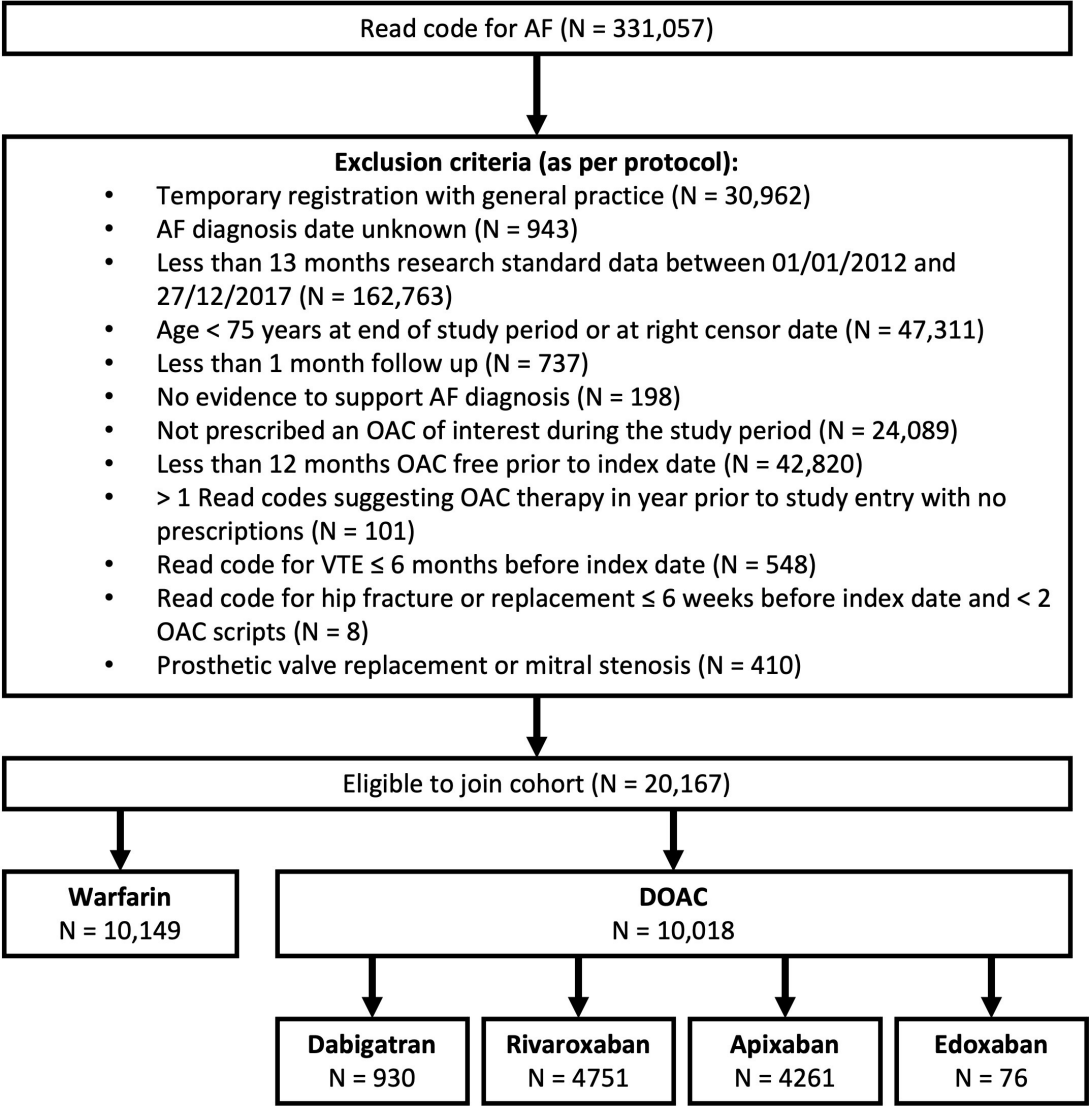
Cohort selection process showing the number of patients excluded at each stage and the number included in each group at study entry. AF, atrial fibrillation; DOAC, direct oral anticoagulant; OAC, oral anticoagulant.

There were 331 057 patients identified with a Read code for AF. Figure 2 shows the number of patients excluded at each stage and the reasons for exclusion. A total of 20 167 patients were eligible to join the cohort. The index oral anticoagulant was warfarin for 10 149 people and a DOAC for 10 018 people. The median time in the study was 1.49 years (IQR 0.71–2.55 years).

During the study, 3940 (19.5%) patients had at least one period where they were unexposed to anticoagulation. A total of 5603 (27.8%) patients had a change in anticoagulant exposure (either from exposed to unexposed or one anticoagulant type to another) with 3772 (18.7%) switching once, 1154 (5.7%) switching twice and 677 (3.3%) switching more than twice. Of everyone who switched (and using their first switch only), 1365 (24.4%) changed from warfarin to being unexposed, 1294 (23.1%) from a DOAC to being unexposed, 1690 (30.2%) switched from warfarin to a DOAC, 1040 (18.6%) from a DOAC to a different DOAC, and 214 (3.8%) from a DOAC to warfarin.

Table 1 describes the baseline demographics, comorbidities and medications for patients assigned to the warfarin or DOAC groups at study entry. While the groups were similar, the DOAC group was slightly older and had a higher percentage of women. More patients in the DOAC group had a history of ischaemic stroke, transient ischaemic attack or thromboembolism, acute kidney injury and bleeding. Dementia, falls and fracture were also more common in the DOAC group. Other covariates were identified as statistically different between groups (eg, CHA_2_DS_2_-VASc, HASBLED) but the mean/risk differences were negligible and unlikely to have a substantial effect on the analyses.

**Table 1:** Baseline demographics, comorbidities and medication prescribed within 3 months of study entry for patients with AF aged ≥75 years, newly started on warfarin or a DOAC. Results are presented as number of patients (%), median (IQR) or mean (SD). Differences between groups are shown as either risk difference for binary variables (eg, comorbidity present or not), median difference (CI and value of p calculated using bootstrapped difference in medians) mean difference (CI and value of p calculated using T-test)

	Warfarin(n=10 149)	DOAC(n=10 018)	Difference (95% CI)	P value
Age in years (IQR)	81 (78–85)	82 (78–87)	1 (0.47 to 1.53)	<0.01
75–79	3777 (37.2)	3273 (32.7)	–	–
80–84	3390 (33.4)	2981 (29.8)	–	–
85–89	2160 (21.3)	2327 (23.2)	–	–
90+	822 (8.1)	1437 (14.3)	–	–
Sex (female)	5043 (49.7)	5219 (52.1)	0.02 (0.01 to 0.04)	<0.01
Current smoker	541 (5.3)	549 (5.5)	0.01 (−0.02 to 0.04)	0.64
Heavy or problem drinker	330 (3.3)	372 (3.7)	0.03 (0.00 to 0.07)	0.07
Weight in kg (IQR)	76 (66–87)	75 (64–86)	1.20 (−1.71 to to -0.69)	<0.01
Mean CHADS-VASC Score (SD)	4.3 (1.4)	4.4 (1.4)	−0.07 (−0.10 to -0.03)	<0.01
Mean HAS-BLED Score (SD)	3.7 (1.3)	3.7 (1.3)	−0.04 (−0.07 to 0.00)	0.05
Comorbidities				
Heart failure	1614 (15.9)	1505 (15.0)	−0.17 (−0.37 to 0.00)	0.08
Diabetes mellitus	2143 (21.1)	2053 (20.5)	−0.01 (−0.03 to 0.01)	0.28
Hypertension	7222 (71.2)	7015 (70.0)	−0.01 (−0.03 to 0.00)	0.08
Ischaemic stroke/transient ischaemic attack/thromboembolism	2113 (20.8)	2469 (24.6)	0.05 (0.04 to 0.07)	<0.01
Coronary artery disease	2727 (26.9)	2540 (25.4)	0.02 (−0.04 to 0.00)	0.01
Peripheral vascular disease	930 (9.2)	954 (9.5)	0.01 (−0.01 to 0.03)	0.38
Chronic renal impairment	3501 (34.5)	3540 (35.3)	0.01 (−0.01 to 0.02)	0.21
Acute kidney injury	868 (8.6)	1234 (12.3)	0.09 (0.07 to 0.10)	<0.01
Liver disease	173 (1.7)	198 (2.0)	0.04 (−0.01 to 0.09)	0.15
Previous bleed (any)	4203 (41.4)	4319 (43.1)	0.02 (0.00 to 0.03)	0.02
Major bleed	1782 (17.6)	1926 (19.2)	0.03 (0.01 to 0.05)	<0.01
Non-major bleed	3125 (30.8)	3187 (31.8)	0.01 (0.00 to 0.03)	0.12
Intracranial	101 (1.0)	163 (1.6)	0.12 (0.06 to 0.18)	<0.01
Gastrointestinal	1585 (15.6)	1638 (16.4)	0.01 (−0.01 to 0.03)	0.16
Other site	4203 (41.4)	4319 (43.1)	0.02 (0.00 to 0.03)	0.02
Dementia	273 (2.7)	677 (6.8)	0.23 (0.20 to 0.26)	<0.01
Median Frailty Score* (IQR)	0.25 (0.19–0.33)	0.25 (0.19–0.33)	0.00 (0.00 to 0.00)	–
Fall in past year	516 (5.1)	729 (7.3)	0.10 (0.07 to 0.12)	<0.01
Fragility fracture	1802 (17.8)	2095 (20.9)	0.05 (0.03 to 0.07)	<0.01
Mean GP encounters in past year (SD)	13.1 (9.6)	13.1 (10.1)	−0.06 (−0.33 to 0.21)	0.66
Medication				
Antihypertensives	8594 (84.7)	8084 (80.7)	−0.07 (−0.09 to -0.05)	<0.01
Antiplatelets	5803 (57.2)	5435 (54.3)	−0.03 (−0.04 to -0.02)	<0.01
Non-steroidal anti-inflammatories	286 (2.8)	281 (2.8)	0.00 (−0.04 to 0.04)	0.96
Corticosteroids	929 (9.2)	856 (8.5)	−0.02 (−0.04 to 0.01)	0.13
Selective serotonin reuptake inhibitors	649 (6.4)	753 (7.5)	0.04 (0.02 to 0.07)	<0.01
Proton pump inhibitor or H2 receptor antagonist	4263 (42.0)	4420 (44.1)	0.02 (0.01 to 0.04)	<0.01
Statins	5284 (52.1)	4989 (49.8)	−0.02 (−0.04 to -0.01)	<0.01
Diabetic medications	1338 (13.2)	1229 (12.3)	−0.02 (−0.04 to 0.00)	0.05
Anticonvulsants	455 (4.5)	591 (5.9)	0.07 (0.04 to 0.10)	<0.01
Antiarrhythmics	373 (3.7)	361 (3.6)	−0.01 (−0.04 to 0.03)	0.79

* The Frailty Score can range from 0 to 1 with higher numbers indicating the most severe frailty. For this study, frailty was calculated using the deficits described in the Electronic Frailty Index (eFI).^25^ The number of deficits present are summed and then divided by 36, frailty is categorised as: 0–0.12 = fit; >0.12–0.24 = mild frailty; >0.24–0.36 = moderate frailty; >0.36 = severe frailty.

AF, atrial fibrillation; DOAC, direct oral anticoagulant; GP, general practice.

Table 2 shows the crude incidence rates for each outcome when unexposed, exposed to any anticoagulant, and by exposure type (warfarin, all DOACs, rivaroxaban, apixaban). Results for dabigatran and edoxaban are not reported due to low numbers of patients. The rates of stroke, myocardial infarction and death were substantially higher in the unexposed patients than either the warfarin or DOAC groups. The rates of major and gastrointestinal bleeding were similar between the exposed and unexposed groups. The rate of intracranial haemorrhage was lower in the exposed group but of borderline statistical significance. Non-major bleeding was higher in the exposed group.

**Table 2:** Incidence rates of outcomes per 1000 person-years for patients with atrial fibrillation aged ≥75 years and unadjusted HRs comparing exposed to unexposed and DOACs to warfarin for each outcome

Drug	Events	Person-time (years)	Incidence per 1000 person-years (95% CI)	Crude HR (95% CI)	P value
**Ischaemic/unspecified stroke**					
Unexposed	92	2820	32.6 (26.6 to 40.0)	Ref	Ref
Exposed	385	31 323	12.3 (11.1 to 13.6)	0.30 (0.24 to 0.38)	<0.01
Warfarin	208	17 576	11.8 (10.3 to 13.6)	Ref	Ref
All DOACs	177	13 748	12.9 (11.1 to 14.9)	1.02 (0.84 to 1.25)	0.81
Rivaroxaban	93	6756	13.8 (11.2 to 16.9)	1.11 (0.87 to 1.41)	0.42
Apixaban	73	5435	13.4 (10.7 to 16.9)	1.05 (0.80 to 1.37)	0.74
**Major bleed**					
Unexposed	84	2598	32.3 (26.1 to 40.0)	Ref	Ref
Exposed	961	30 816	31.2 (29.3 to 33.2)	0.88 (0.70 to 1.10)	0.25
Warfarin	493	17 237	28.6 (26.2 to 31.2)	Ref	Ref
All DOACs	468	13 579	34.5 (31.5 to 37.7)	1.15 (1.02 to 1.31)	0.03
Rivaroxaban	269	6645	40.5 (35.9 to 45.6)	1.36 (1.18 to 1.58)	<0.01
Apixaban	145	5421	26.8 (22.7 to 31.5)	0.88 (0.73 to 1.06)	0.18
**Non-major bleed**					
Unexposed	82	2606	31.5 (25.3 to 39.1)	Ref	Ref
Exposed	1817	29 476	61.6 (58.9 to 64.5)	1.73 (1.38 to 2.16)	<0.01
Warfarin	977	16 430	59.5 (55.9 to 63.3)	Ref	Ref
All DOACs	840	13 046	64.4 (60.2 to 68.9)	1.03 (0.94 to 1.13)	0.57
Rivaroxaban	518	6299	82.2 (75.5 to 89.6)	1.32 (1.19 to 1.47)	<0.01
Apixaban	251	5269	47.6 (42.1 to 53.9)	0.74 (0.65 to 0.86)	<0.01
**Intracranial bleed**					
Unexposed	17	2825	6.0 (3.7 to 9.7)	Ref	Ref
Exposed	114	31 792	3.6 (2.9 to 4.3)	0.58 (0.35 to 0.97)	0.04
Warfarin	66	17 787	3.7 (2.9 to 4.7)	Ref	Ref
All DOACs	48	14 004	3.4 (2.6 to 4.6)	0.92 (0.63 to 1.34)	0.66
Rivaroxaban	26	6870	3.8 (2.6 to 5.6)	1.02 (0.65 to 1.61)	0.93
Apixaban	20	5565	3.6 (2.3 to 5.6)	0.96 (0.58 to 1.59)	0.88
**Gastrointestinal bleed**					
Unexposed	64	2700	23.7 (18.6 to 30.3)	Ref	Ref
Exposed	781	30 955	25.2 (23.5 to 27.1)	0.94 (0.72 to 1.21)	0.61
Warfarin	390	17 292	22.6 (20.4 to 24.9)	Ref	Ref
All DOACs	391	13 663	28.6 (25.9 to 31.6)	1.21 (1.05 to 1.39)	0.01
Rivaroxaban	219	6688	32.8 (28.7 to 37.4)	1.39 (1.18 to 1.64)	<0.01
Apixaban	121	5444	22.2 (18.6 to 26.6)	0.92 (0.75 to 1.13)	0.41
**Myocardial infarction**					
Unexposed	49	2843	17.2 (13.0 to 22.8)	Ref	Ref
Exposed	296	31 467	9.4 (8.4 to 10.5)	0.47 (0.35 to 0.64)	<0.01
Warfarin	162	17 585	9.2 (7.9 to 10.7)	Ref	Ref
All DOACs	134	13 882	9.7 (8.2 to 11.4)	1.00 (0.79 to 1.25)	0.972
Rivaroxaban	54	6820	7.9 (6.1 to 10.3)	0.82 (0.61 to 1.12)	0.22
Apixaban	64	5502	11.6 (9.1 to 14.9)	1.18 (0.88 to 1.57)	0.27
**Death**					
Unexposed	795	2917	272.5 (254.2 to 292.1)	Ref	Ref
Exposed	2388	31 847	75.0 (72.0 to 78.1)	0.28 (0.26 to 0.31)	<0.01
Warfarin	1185	17 816	66.5 (62.8 to 70.4)	Ref	Ref
All DOACs	1203	14 031	85.7 (81.0 to 90.7)	1.30 (1.20 to 1.41)	<0.01
Rivaroxaban	610	6881	88.7 (81.9 to 96.0)	1.34 (1.22 to 1.48)	<0.01
Apixaban	478	5576	85.7 (78.4 to 93.8)	1.30 (1.17 to 1.45)	<0.01

DOAC, direct oral anticoagulant.

The incidence of stroke was similar between warfarin and DOACs. The incidence of major and gastrointestinal bleeding was higher with DOACs as a group than warfarin, however apixaban had lower and rivaroxaban a higher incidence of all three outcomes than warfarin when analysed separately. Rates of intracranial haemorrhage and myocardial infarction were similar but had low numbers of events. The rate of death was higher in the DOAC groups than warfarin.

**Figure 3:**
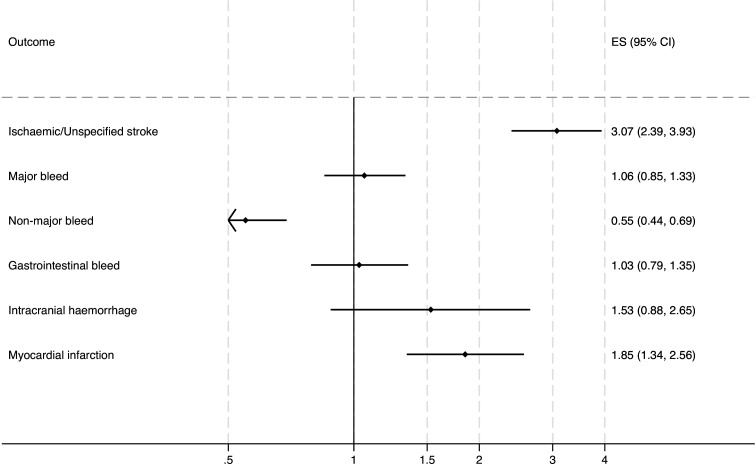
HRs for effectiveness and safety outcomes associated when not exposed to an anticoagulant compared with exposed to any anticoagulant from the Cox proportional hazards model adjusted for age, sex and relevant comorbidities. ES, effect size.

Figure 3 shows the adjusted HRs for effectiveness (stroke) and safety (bleeding) outcomes when exposed to anticoagulation compared with unexposed.

The risk of adverse health events was significantly increased when unexposed compared with time exposed to any anticoagulation. The risk of ischaemic and unspecified stroke increased (HR 3.07, 95% CI 2.49 to 4.02), as did the risk of death (HR 2.87, 95% CI 2.63 to 3.12). However, for death, the simple model violated the proportional hazards assumption so exposure was subsequently allowed to interact with time. In the time-varying model, the risk of death during unexposed periods was similar to the simple model at time 0 (HR 3.16, 95% CI 2.73 to 3.65) and did not change significantly over time. The risk of myocardial infarction almost doubled (HR 1.85, 95% CI 1.34 to 2.56) during unexposed periods.

The median time to death for those who died in an unexposed period was 169 days (IQR 54–437) from the end of the last exposed period. For ischaemic and unspecified stroke, the median time was 182 days (IQR 62–419). For myocardial infarction, the median time was 165 days (IQR 57–375).

The post hoc analysis did not support the hypothesis that treatment gaps or cessation were due to end-of-life care. In the top 100 Read codes in the month before death, only 15 of the 795 people who died in an unexposed period had a Read code for palliative care input or referral to palliative care. The most common Read codes were those associated with general practice (GP) contact (eg, a telephone encounter, advice or a home visit) or a hospital contact (eg, a discharge summary, seen in hospital casualty, seen in clinic) but there were no Read codes in the top 100 for serious bleeds, stroke or myocardial infarction.

**Figure 4:**
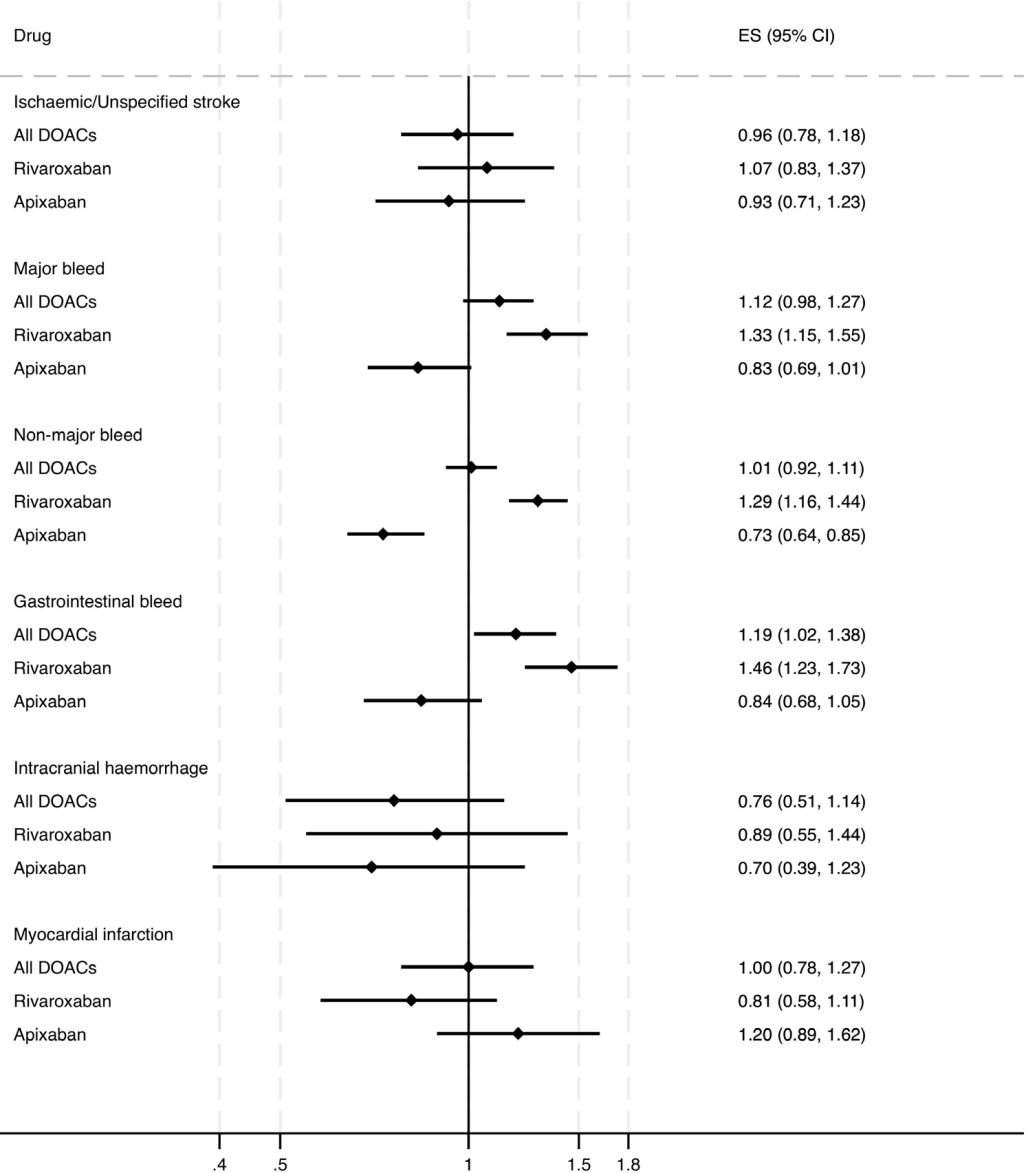
HRs for effectiveness and safety outcomes associated with all DOAC, rivaroxaban and apixaban compared with warfarin from the Cox proportional hazards model adjusted for age, sex and relevant comorbidities. DOAC, direct oral anticoagulant; ES, effect size.

Figure 4 shows the adjusted HRs for effectiveness (stroke) and safety (bleeding) outcomes for all DOACs, rivaroxaban, apixaban compared with warfarin.

When DOACs as a class were compared with warfarin, there was no difference in effectiveness (HR 0.96, 95% CI 0.78 to 1.18). Major (HR 1.12, 95% CI 0.98 to 1.27) and non-major bleeding risks (HR 1.01, 95% CI 0.92 to 1.11) were also similar, however DOACs were associated with a 20% increase in the risk of gastrointestinal bleeding compared with warfarin (HR 1.19, 95% CI 1.02 to 1.38). There were no significant differences between any of the groups and warfarin for intracranial haemorrhage but there were few events for this outcome (n=114). The risk of death was slightly increased with DOACs compared with warfarin (HR 1.14, 95% CI 1.05 to 1.25) in the simple model, but this violated the proportionality assumption and when exposure was allowed to interact with time the risk of death was no longer significantly increased (HR 1.05, 95% CI 0.92 to 1.21) at time 0 and did not increase significantly over time.

When apixaban and rivaroxaban were analysed individually, rivaroxaban had a 30% higher risk of major (HR 1.33, 95% CI 1.15 to 1.55) and non-major (HR 1.29, 95% CI 1.16 to 1.44) bleeding and a 46% increase in risk of gastrointestinal bleeding (HR 1.46, 95% 1.23 to 1.73) than warfarin. Apixaban had a 27% lower risk (HR 0.73, 95% CI 0.64 to 0.85) of non-major bleeds, and was borderline for a lower risk of major bleeds (HR 0.83, 95% CI 0.69 to 1.01) than warfarin, there was no significant difference in gastrointestinal bleeding (HR 0.84, 95% CI 0.68 to 1.05). Neither apixaban (HR 1.01, 95% CI 0.84 to 1.21) nor rivaroxaban (HR 1.14, 95% CI 0.96 to 1.35) were associated with a significant change in the risk of death compared with warfarin. Sensitivity analyses using an intention-to-treat approach showed similar results (online supplemental appendix 2).

## Discussion

This study is the first to evaluate safety and effectiveness outcomes of anticoagulants in older people accounting for changes in treatment over time, including periods where anticoagulant therapy is discontinued or withheld and subsequently restarted. The risks of stroke and death were three times higher during unexposed periods, and the risk of myocardial infarction was almost double that of anticoagulant treated periods. The risk of major bleeds was not significantly reduced in the unexposed periods compared with time on anticoagulant treatment, but the risk of non-major bleeds was lower. There was no evidence that DOACs as a group were any different to warfarin in any of the studied outcomes except for a slight increase in the risk of gastrointestinal bleeding. However, when apixaban and rivaroxaban were analysed separately, rivaroxaban had a higher risk of bleeding (major, non-major and gastrointestinal) whereas apixaban showed a lower risk of the same bleed types than warfarin.

The increased risk of serious events when anticoagulation is withheld or discontinued in older patients is concerning, especially as stopping therapy appears to have little effect on reducing the risk of major bleeding, which is often a reason for these agents to be stopped. Our results are similar to those seen in the Global Anticoagulant Regsitry in the FIELD-Atrial Fibrillation (GARFIELD-AF) registry after discontinuation of anticoagulation (stroke/systemic embolism: HR 2.21, 95% CI 1.42 to 3.44; myocardial infarction: HR 1.85, 95% CI 1.09 to 3.13; death: HR 1.62, 95% CI 1.25 to 2.09), however it should be noted that their patients were much younger than our cohort with only a third being aged ≥75 years.^20^ Ischaemic stroke has been shown to double in older patients both in the UK and Denmark (HR 2.99, 95% CI 2.31 to 3.86, UK and HR 2.30, 95% CI 1.79 to 2.95, Denmark) who discontinued anticoagulation.^18^ Despite using a different UK data set and a nested case-control design, the authors found a very similar result thus strengthening the likelihood that this increase in stroke is a true risk of stopping anticoagulant therapy.

The difference in bleeding risk between the individual DOACs is a finding also observed in subgroup analyses of the main randomised controlled trials (ROCKET-AF and ARISTOTLE) which showed rivaroxaban had higher, and apixaban lower bleeding risks than warfarin in older patients.^28 29^ Recent observational studies have largely supported these findings: Kim and colleagues from the USA and Rutherford and colleagues from Norway reported similar safety results to those reported in our study. Apixaban was associated with less major bleeding in both studies (HR 0.51, 95% CI 0.46 to 0.55; sub-HR 0.74, 95% CI 0.60 to 0.91, respectively)^30 31^ and less or similar gastrointestinal bleeding risk (HR 0.52, 95% CI 0.46 to 0.58; sub-HR 0.92, 95% CI 0.75 to 1.22, respectively)^30 31^ than warfarin. Rivaroxaban had higher rates of major bleeding (HR 1.09, 95% CI 1.03 to 1.17) in the US study^30^ but was similar to warfarin in the Norwegian study (sub-HR 0.96, 95% CI 0.78 to 1.16),^31^ however major gastrointestinal bleeding was higher with rivaroxaban than warfarin in both studies (HR 1.40, 95% CI 1.29 to 1.52; sub-HR 1.43, 95% CI 1.19 to 1.73, respectively).^30 31^

One study from France found that the risk of major bleeding was significantly lower with rivaroxaban than warfarin (HR 0.53, 95% CI 0.33 to 0.85),^32^ however this study recruited patients that had been on therapy for up to 6 months rather than new users, which may bias the results towards adherent or tolerant users and it ignores early events. Both apixaban (HR 0.51, 95% CI 0.43 to 0.60) and rivaroxaban (HR 0.62, 95% CI 0.53 to 0.72) were associated with a lower risk of intracranial haemorrhage in the US study,^30^ which is in keeping with the results of the pivotal randomised controlled trials.^28 29^ Our study found no evidence of a difference in intracranial haemorrhage (ICH) risk between either apixaban or rivaroxaban and warfarin; the Norwegian study found the same (apixaban: sub-HR 0.67, 95% CI 0.44 to 1.31; rivaroxaban: sub-HR 0.79, 95% CI 0.55 to 1.13).^31^ This is likely because of the low number of ICH events that occurred in both studies. Another US study found no difference in any bleeding outcomes with apixaban or rivaroxaban compared with warfarin, however, this is likely due to the small number of events in each group.^33^

Making comparisons between treated and untreated patients is not recommended in cohort studies as it can lead to various types of bias, therefore an ‘active-comparator, new-user’ study design is advocated. The major strength of this study is that it employs an active-comparator design and anchors the start of exposure at initiation of anticoagulant treatment. However, unlike previous studies which censor patients at the point of switching or discontinuation of therapy,^30 31 33^ exposure to anticoagulation was mapped over time, allowing patients to switch between treatments and events are included which occur in unexposed periods. This enabled us to gain a more complete picture of how different treatments and non-treatment can impact both safety and effectiveness outcomes in the older population. One limitation of this method is that because exposure is estimated there may be some problems with temporality if the outcome is the cause of oral anticoagulant cessation, but it appears that the event occurred during a period of non-exposure. However, it will also capture events in people who stopped therapy due to side effects or who developed contraindications to treatment, which is more akin to real-life treatment than censoring. After reviewing the primary results, we conducted a post hoc analysis to investigate whether the increased risk of death observed in unexposed periods could be due to medication being stopped for palliation; we found no evidence to support this hypothesis. The use of more complex causal inference methods such as G-estimation or marginal structural methods would enable us to account for time-varying confounding in addition to time-varying exposure. This may be useful in this cohort as previous treatment and adverse effects such as bleeding can affect future treatments.

The results of this study are relevant to all clinicians treating older patients with AF when making decisions on whether to prescribe or deprescribe anticoagulants. It has been shown that anticoagulation is still underused in older people, and that a history of falls, dementia and advancing age are all associated with a decrease in prescribing.^14^ Prescribers might be unconvinced that the evidence of benefit with anticoagulation outweighs the risk of bleeding in these patients,^11^ and anticoagulation is often deprescribed for the same reasons. Addressing modifiable risk factors for stroke and bleeding (eg, smoking, alcohol consumption and blood pressure control) may make anticoagulation safer and also reduce stroke risk independent of treatment. There is a need to focus not only on comparing different anticoagulant strategies to ensure the safest and most effective treatments are used, but also to examine the risks of deprescribing anticoagulants so that the risks of doing so can be adequately discussed with patients before coming to a shared treatment decision.

## Supplementary material

10.1136/heartjnl-2024-324763 online supplemental file 1

## Data Availability

Data may be obtained from a third party and are not publicly available.
