# Supplementary material for: Safety and effectiveness of anticoagulation therapy in older people with atrial fibrillation during exposed and unexposed treatment periods
Source: Heart. 2025 Feb 17;111(12):e324763. doi: 10.1136/heartjnl-2024-324763 (PMC12171496; doi:10.1136/heartjnl-2024-324763)

Comparison of safety and effectiveness outcomes when exposed and unexposed to anticoagulation therapy for stroke prevention in people aged ≥ 75 years with atrial fibrillation: a retrospective cohort study

Appendix 1: Supplementary methods

Exclusion criteria

Patients were excluded if they had a Read code for venous thromboembolism, in the 6 months prior to the index date; had a Read code for hip fracture or hip replacement in the 6 weeks prior to the index date; or had a Read code for mitral stenosis or prosthetic heart valve replacement at any time prior to the index date.

Exposure

Exposure was defined as at least one prescription issued for one of the anticoagulants of interest (warfarin any strength, dabigatran 110mg or 150mg, rivaroxaban 15mg or 20mg, apixaban 2.5mg or 5mg, edoxaban 30mg or 60mg). Prescriptions for other strengths of DOAC not licensed for stroke prevention in AF were excluded.

Anticoagulant exposure was mapped from the index date to the study exit date and patients were allowed to switch between treatments. Patients could contribute time to both the DOAC and warfarin groups or be unexposed if there was a gap of > 60 days between consecutive DOAC prescriptions; for warfarin, patients were unexposed if they had more than twice their median prescription gap calculated from their prior prescriptions. The algorithm for mapping warfarin has been described previously, patients were classed as unexposed if they had more than twice their median prescription gap calculated from their prior prescriptions

Comorbidities

Comorbidities assessed at all time before index date: heart failure, hypertension, diabetes mellitus, renal disease, liver disease, dementia, coronary artery disease, peripheral vascular disease, stroke, transient ischaemic attack, thromboembolism, bleeding, falls, number of general practice encounters in the year preceding the index date.

Appendix 2: Hazard ratios for effectiveness and safety outcomes in as-treated and intention-to-treat analyses associated with all DOAC, rivaroxaban, apixaban and unexposed periods compared with warfarin from the Cox proportional hazards model adjusted for age, sex, and relevant comorbidities. HR = hazard ratio, CI = confidence interval

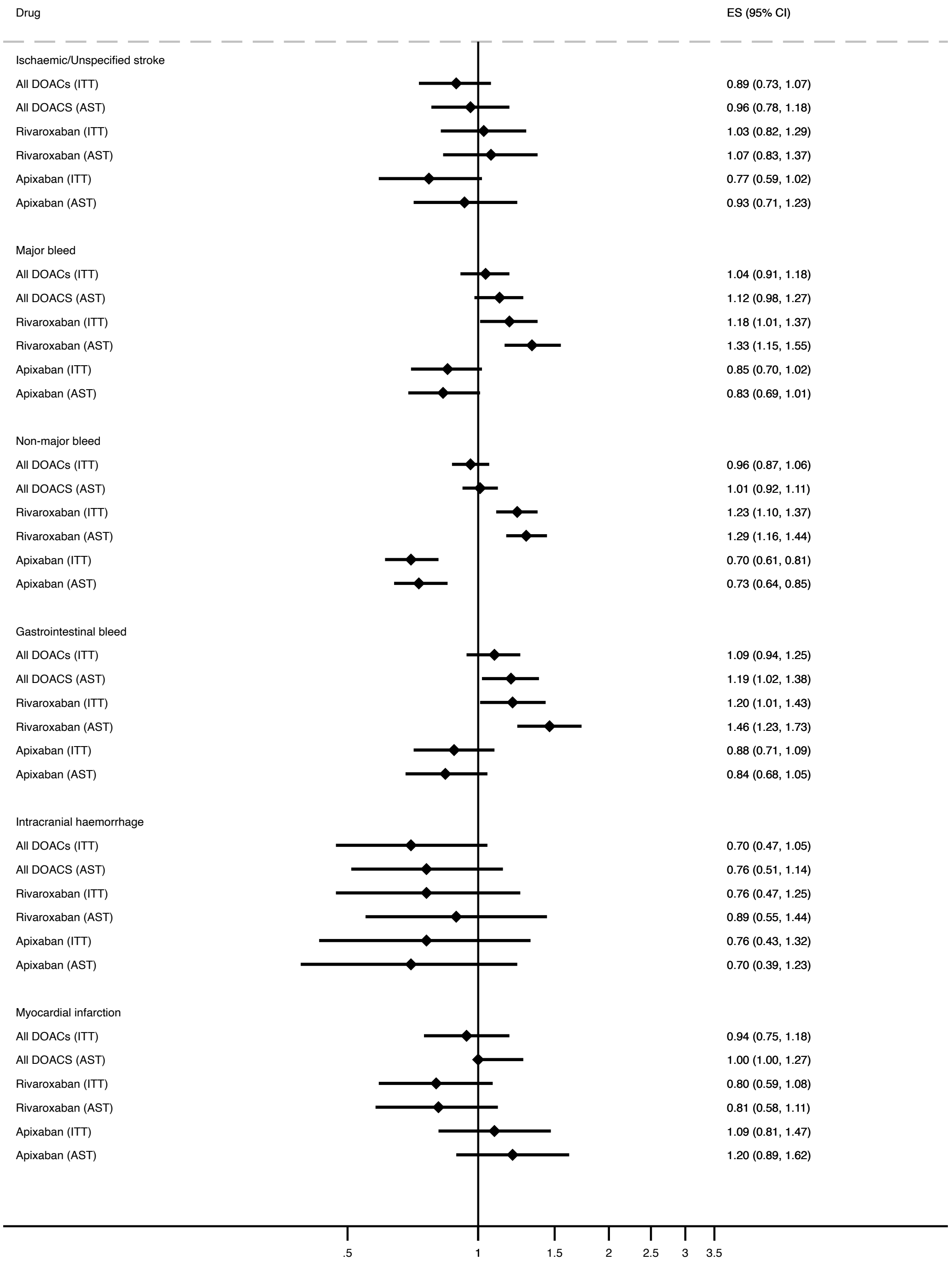

Supplement: online supplemental file 1 [file heartjnl-111-12-s001.pdf]
